# Supplementary material for: Comparative study on the changes of bacterial species and severity of antimicrobial resistance during 13 years
Source: PLoS One. 2021 Aug 25;16(8):e0256375. doi: 10.1371/journal.pone.0256375 (PMC8386881; doi:10.1371/journal.pone.0256375)
Supplement: Attachment — Submitted filename: the details of the professional service.docx [file pone.0256375.s001.docx]

Dear editors,

We have edited our manuscript with the AJE which PLOS ONE recommended, thoroughly copyedit our manuscript for language usage, spelling, and grammar.

Thank you for your time.
