# Supplementary material for: Comparative study on the changes of bacterial species and severity of antimicrobial resistance during 13 years
Source: PLoS One. 2021 Aug 25;16(8):e0256375. doi: 10.1371/journal.pone.0256375 (PMC8386881; doi:10.1371/journal.pone.0256375)
Supplement: Attachment — Submitted filename: Response to Reviewers.docx [file pone.0256375.s002.docx]

Dear Editors and Reviewers:

Thank you for your letter and for the reviewers’ comments concerning our manuscript titled “Comparative study on the changes of bacterial species and severity of antimicrobial resistance during 13 years”(PONE-D-21-10409).The comments were very valuable and helpful in revising and improving our paper; they also provided significant guidance in discussing our research. We studied your comments carefully and have made many corrections to our manuscript, which we hope will be considered favourably. The major corrections to our original paper and our responses to the reviewers’ comments follow below.

**Answer to Reviewers:**

**Response to 1:** The manuscript is technically sound and the data support the conclusions.

**Response to 2:** The statistical analysis has been performed appropriately and rigorously.

**Response to 3:** The authors have made all data underlying the findings in their manuscript fully available.

**Response to 4:** The manuscript is presented in an intelligible fashion and written in standard English. We have edited our manuscript with the AJE which PLOS ONE recommended, thoroughly copyedit our manuscript for language usage, spelling, and grammar.

**Response to 5:** We have revised language editing and grammar mistakes in the manuscript. The scientific names of bacterial pathogens were writen in italic form.

1) Title: We have modify the title.
2) Abstract: We have revised the abstract to include the main conclusions of the study.
3) Introduction: We have added the virulence determinants, the pathogenesis, and diseases caused by S. aureus, E. coli, Acinetobacter baumannii, and K. pneumonia in instroduction. We added paragraphs given by reviewers to the manuscript and cited references. The purpose of the study was restated.
4) Material and methods: The data on the origin of the specimen, due to the loss of data 13 years ago, only data from 2018 to 2020, so it is not listed in the manuscript. The data of the last two years is shown in the following tables.

| Age | Number of people | Male | Female |
| --- | --- | --- | --- |
| <18 | 109 | 70 | 39 |
| 19-59 | 819 | 524 | 295 |
| ≧60 | 2028 | 1146 | 882 |
| Totle | 2956 | 1740 | 1216 |
|  |  |  |  |
|  |  |  |  |
| Department | Number of cases | Department | Number of cases |
| ICU | 14 | Burns department | 260 |
| Wound repair department | 225 | Neurology department | 288 |
| Pediatric department | 39 | Neurosurgery department | 380 |
| Otolaryngology department | 11 | Nephropathy Rheumatology department | 155 |
| Obstetrics and gynecology department | 11 | Pain management | 4 |
| Orthopedics department | 144 | Gastroenterology department | 243 |
| Pneumology department | 615 | Department of Cardiology | 56 |
| Urinary surgery | 42 | Oncology department | 60 |
| Endocrinology department | 109 | Ophthalmology department | 7 |
| General surgery department | 35 | Hematology department | 77 |
| General medical department | 183 |  |  |
| Totle |  |  | 2956 |

5) The subtitle: Bacteria isolation and identification have modified to be: Isolation and identification of bacterial pathogens. We have added the company names and countries of the used bacterial media. The biochemical reactions according to instructions. We have update the CLSI reference use 2018 version. Multilocus sequence typing it's not applicable in our study.

6) Results: The scientific names of bacterial pathogens were in italic form.

7) Discussion: We have revised the discussion as requested.

8) Conclusion: We have revised the discussion as requested.

**Response to 6:** No peer review of published articles.

Once again, thank you very much for your comments and suggestions; we hope that our revised manuscript is of the quality that is worthy of your journal.

Sincerely yours,

Huili Zhang
